# CT-based machine learning radiomics predicts CCR5 expression level and survival in ovarian cancer

**DOI:** 10.1186/s13048-022-01089-8

**Published:** 2023-01-03

**Authors:** Sheng Wan, Tianfan Zhou, Ronghua Che, Ying Li, Jing Peng, Yuelin Wu, Shengyi Gu, Jiejun Cheng, Xiaolin Hua

**Affiliations:** 1 Department of Gynecology and Obstetrics, Shanghai Key Laboratory of Maternal Fetal Medicine, Shanghai Institute of Maternal-Fetal Medicine and Gynecologic Oncology, Shanghai First Maternity and Infant Hospital, School of Medicine, Tongji University, Shanghai, 200092 China; 2 Shanghai Key Laboratory of Maternal Fetal Medicine, Shanghai Institute of Maternal-Fetal Medicine and Gynecologic Oncology, Shanghai First Maternity and Infant Hospital, School of Medicine, Tongji University, Shanghai, 200092 China; 3 Reproductive Medicine Center, Tongji Hospital Affiliated to Tongji University, Shanghai, China; 4 Department of Radiology, Shanghai Key Laboratory of Maternal Fetal Medicine, Shanghai Institute of Maternal-Fetal Medicine and Gynecologic Oncology, Shanghai First Maternity and Infant Hospital, School of Medicine, Tongji University, Shanghai, 200092 China; 5 Department of Radiology, Shanghai First Maternity and infant hospital, Shanghai Tongji University School of Medicine, 2699 West Gaoke Road, Shanghai, 201204 China; 6 Department of Obstetrics, Shanghai First Maternity and infant hospital, Shanghai Tongji University School of Medicine, 2699 West Gaoke Road, Shanghai, 201204 China

**Keywords:** CCR5, Ovarian cancer, Radiomics, Machine learning, Prognosis, Survival

## Abstract

**Objective:**

We aimed to evaluate the prognostic value of C-C motif chemokine receptor type 5 (CCR5) expression level for patients with ovarian cancer and to establish a radiomics model that can predict CCR5 expression level using The Cancer Imaging Archive (TCIA) and The Cancer Genome Atlas (TCGA) database.

**Methods:**

A total of 343 cases of ovarian cancer from the TCGA were used for the gene-based prognostic analysis. Fifty seven cases had preoperative computed tomography (CT) images stored in TCIA with genomic data in TCGA were used for radiomics feature extraction and model construction. 89 cases with both TCGA and TCIA clinical data were used for radiomics model evaluation. After feature extraction, a radiomics signature was constructed using the least absolute shrinkage and selection operator (LASSO) regression analysis. A prognostic scoring system incorporating radiomics signature based on CCR5 expression level and clinicopathologic risk factors was proposed for survival prediction.

**Results:**

CCR5 was identified as a differentially expressed prognosis-related gene in tumor and normal sample, which were involved in the regulation of immune response and tumor invasion and metastasis. Four optimal radiomics features were selected to predict overall survival. The performance of the radiomics model for predicting the CCR5 expression level with 10-fold cross- validation achieved Area Under Curve (AUCs) of 0.770 and of 0.726, respectively, in the training and validation sets. A predictive nomogram was generated based on the total risk score of each patient, the AUCs of the time-dependent receiver operating characteristic (ROC) curve of the model was 0.8, 0.673 and 0.792 for 1-year, 3-year and 5-year, respectively. Along with clinical features, important imaging biomarkers could improve the overall survival accuracy of the prediction model.

**Conclusion:**

The expression levels of CCR5 can affect the prognosis of patients with ovarian cancer. CT-based radiomics could serve as a new tool for prognosis prediction.

**Supplementary Information:**

The online version contains supplementary material available at 10.1186/s13048-022-01089-8.

## Introduction

Ovarian cancer is one of the most common gynecological malignant tumors worldwide, with more than 10,000 patients suffering from it every year [1]. The fatality rate of ovarian cancer ranks high among gynecological malignancies, and it carries a dismal prognosis. Despite remarkable achievements in treating ovarian cancer, such as debulking surgery, chemotherapy, immunotherapy, and targeted therapy, its 5-year survival rate is only approximately 30% [2]. Intratumor heterogeneity within patients and inter-tumor heterogeneity among patients present significant challenges for predicting overall survival and effectiveness of therapy. Although clinicopathologic features, serum markers including cancer antigen 125, and traditional imaging findings have served as prognostic indicators, limitations like low specificity [3] and requirement for surgical specimens and professional staff make them difficult to meet the clinical need. Thus, the search for additional reliable prognostic indicators continues to enable personalized precision medicine.

The tumor microenvironment, which involves multiple immune infiltrations, plays an essential role in the prognosis and clinical benefit of therapy. The chemokines and their specific receptors could mediate migration and impact the cellular process of both tumor and immune cells [4, 5]. The C-C motif chemokine receptor type 5 (CCR5) is one of the G protein-coupled receptors on leukocyte surfaces and is involved in the process of host immune response, which serves as our main defense against pathogens. Emerging evidence indicates CCR5 contributes largely to the tumor invasion and metastasis [6]. High expression of CCR5 causes tumor cell migration and vascular invasion, which can result in distant metastasis of several types of tumors such as breast cancers [7–9], hepatocellular carcinoma [10] and prostate cancers [11–13]. Given its prominence in the regulation of immunity， several clinical trials targeting CCR5 are underway, involving cancers [7, 13], COVID-19 [14], immune deficiency diseases like HIV [15–18], etc. It has been reported that in comparison to CD133^-^ non-cancer stem-like cells (CSLCs), chemokine CCL5 and its receptors, CCR1, CCR3, and CCR5, were significantly increased in CSLCs, and the blocking of CCL5 and its receptors effectively inhibits their invasive capacity [19]; CCR5 were consistently upregulated in Tregs in a patient who had been diagnosed with ovarian cancer， which indicated ovarian CSCs recruit Tregs via CCL5–CCR5 interactions [20].However, to the best of our knowledge, non-invasive and effective methods with a universal prognostic guidance to detect the marker for survival are still lacking.

Preoperative computed tomography (CT) images can tailor the surgery strategy since a wealth of cancer information can be obtained. However, intraoperative exploration and postoperative pathological diagnosis are more widely applied for treatment guidance and response assessment. It is developing an accurate and safe method to evaluate survival and guide treatment before operation remains a significant challenge. With the development of science and technology that enables the digital medical image to be converted into high-throughput data, radiomics has evolved rapidly [21, 22]. Radiomics involves the application of advanced computational analyses of images, increasing visual assessment by extracting features not perceptible to the naked human eye [23]. Combined with machine learning techniques, radiomics is amenable to good performance in cancer prognosis prediction [24]. It provides highly accurate, dynamic and non-invasive methods for personalized medicine. Prior studies have demonstrated that CT imaging features are associated with angiogenesis, metabolism, hypoxia, and microenvironment of cancers [25–27]. Radiomics on CT imaging can be used in early diagnosis, risk stratification, evaluation of residual lesions, and tumor heterogeneity in a patient with ovarian cancer [28–33]. Despite recent advances in radiomics, to our best knowledge, no study involving survival analysis of patients with ovarian tumors has used radiomics features to predict the expression levels of CCR5 and evaluate its prognostic value.

Our current research aims to investigate the association between CCR5 expression levels and the prognosis of patients with ovarian cancer by using data from the Cancer Genome Atlas (TCGA) and matched patient data from the Cancer Imaging Archive (TCIA). Furthermore, based on CT images, we attempted to develop a radiomics model to predict the expression level of CCR5 which could be offered as a reference for clinical decisions.

## Methods

### Data retrieval and processing for this study

343 OC cases from the TCGA portal system (https://portal.gdc.cancer.gov/) were used for the gene-based prognostic analysis. Fifty-seven cases had preoperative CT images stored in TCIA (https://wiki.cancerimagingarchive.net/display/Public/TCGA-LGG) with genomic data in TCGA were used for radiomics feature extraction and model construction. Eighty-nine cases with TCGA and TCIA clinical data were used for radiomics model evaluation. All the samples from TCIA and TCGA were anonymized and publicly available.

The exclusion criteria were: samples with incomplete clinical data without CCR5 expression data; samples without CT images; samples with poor image quality, and images with no corresponding clinical or gene expression information (The brief inclusion/exclusion criteria can be seen in supplemental table 1).

### Identification of CCR5 as a differentially expressed gene

We calculate the cutoff value of CCR5 expression using the survMisc package of R. An online tool (https://www.xiantao.love/products) was employed to characterize the gene expression profiles of different tissues. RNA-seq data in TPM format were uploaded and transferred. Kaplan–Meier (KM) curves and log-rank test were used to calculate survival probability and median survival time. We further performed conditional landmark analyses separately at 12, 36, and 60 months to compare two groups using the package jskm and survival.

Univariate and multivariate analysis of various factors that affect OS was performed by Cox regression. The time-dependent receiver operating characteristic (ROC) analysis was applied to validate the CCR5 expression-based prognostic signature. The relationships between the CCR5 expression and clinical parameters were assessed using the Spearman correlation test.

CIBERSORTx (https://cibersortx.stanford.edu/) was employed to analyze the immune composition based on the gene expression profiles of complex tissues. RNA-seq data in TPM format were uploaded as the mixture file. Impute Cell Fractions, and LM22 (22 immune cell types) were selected for the signature matrix file. Gene ontology (GO) and KEGG enrichment analyses of differentially expressed genes were carried out with the clusterProfiler package in R.

### Radiomics feature extraction and model establishment

The image processing was shown as a supplemental figure 1. Device information and scan parameters are as follows:

- Manufacturer: GE, SIEMENS
- X-ray tube current: median 259.0 mA
- X-ray tube voltage: median 120 kVp
- Slice thickness: median 5.0mm
- Exposure_Times: median 828ms
- Pixel spacing: median 0.742×0.742mm2
- Contrast: Optiray 350, Isovue 370

Before feature selection, data were centered and scaled, implemented by the preProcess function from the R caret (Classification And Regression Training) package. As explained and reported in previous research [34], a Principal Component Analysis (PCA) was performed on the extracted features to plot data in a space of reduced dimensions (Supplemental Figure 8). The radiomics features of the two devices were similar. Radiomics features were extracted from 3D VOIs using Pyradiomics. Spatial Resampling: 1 × 1 × 1 mm3; Intensity Rescaling: 500; Intensity Discretization: binWidth 25 [35, 36]. The region of interest (ROI) delineating each tumor was manually drawn under the supervision of two experienced radiologists. The robustness of the radiomics features was assessed using the intra-class correlation coefficient (ICC) by the R package irr. ICC ≥ 0.75 indicated high consistency, 0.51–0.74 middle, and < 0.5 low. Extracted radiomics features with an ICC of ≥0.75 met the criteria for further analysis, while the others were excluded from the final feature dataset. The least absolute shrinkage and selection operator (LASSO) regression, suitable for high-dimensional data, was performed to select the most useful predictive features using the “glmnet” package of R from the primary data set. Wilcoxon test analyses were used for exploring the relationship between CCR5 expression and radiomics parameters, and the results were visualized using the R package Ggpubr. The predictive radiomics characteristics screened from LASSO regression were incorporated into a multivariate Logistic regression to establish a radiomics model.

### Model evaluation and clinical application

Restricted cubic spline (RCS) was performed using the “rms” and “survMisc” package of R. RCS shows the hazard ratio (HR) and 95% confidence interval (CI) describing the association of Radiomics scores (RS) to allow nonlinear assessment. Clinical and image data of 89 cases of ovarian cancer were retrospectively analyzed in TCIA. The subjects were divided into the low-expression CCR5 and high-expression groups according to Radiomics Score (RS) when the cutoff was 0.388. All patients were divided into the training and validation groups according to a ratio of 7:3.

With the R package of rms, the clinical features and RS were incorporated into multivariate stepwise logistic regression with minimum AIC (Akaike Information Criterion) method to measure goodness of fit. Based on the factors in the final model, a predictive nomogram was generated to predict the 1-year, 3- year, and 5-year prognosis for patients with ovarian cancer. The time-dependent Receiver Operating Characteristic (ROC) curve was plotted. The calibration curve was used to describe and evaluate the nomogram performance visually. The perfect prediction should fall on the diagonal line of the figure.

### Statistical analysis

We conducted all the statistical analysis using R version 3.5 (https://www.r-project.org). All p values < 0.05 was considered statistically significant. The performance of the radiomics model was assessed by using several indices with 10-fold cross-validation on the training and validation set, including accuracy (ACC), specificity (SPE), sensitivity (SEN), positive predictive value (PPV), and negative predictive value (NPV). A ROC curve was also employed to assess the overall performance of the radiomics models, and a Precision-Recall (PR) curve was displayed for comprehensive evaluations of the performance. The area under the curve (AUC) was calculated. We also calculated the Brier score to quantify the radiomics model’s performances. Finally, the decision curve analysis (DCA) was conducted to reveal the clinical usefulness of the radiomics evaluation. Wilcoxon test analyses were used for exploring the relationship between RS and CCR5 expression, and the results were visualized using the R package Ggpubr.

## Results

### Identification of CCR5 as a differentially expressed gene

#### Clinical characteristics of the population with high and low CCR5 expression group

0.829 was calculated as the cutoff of the CCR5 expression. All the cases were divided into high- and low-expression groups according to this value. Comparisons of the clinical characteristics are shown in Table 1. No statistical difference found in clinical factors between two groups, except for age.

**Table 1:** Clinical characteristics of the population with high and low CCR5 expression group

Variables	Total (n = 343)	High (n = 179)	Low (n = 164)	p
Age, n (%)				
<65	220 (64)	124 (69)	96 (59)	0.05
≥65	123 (36)	55 (31)	68 (41)	
Chemotherapy, n (%)				
NO	22 (6)	14 (8)	8 (5)	0.373
YES	321 (94)	165 (92)	156 (95)	
FIGO, n (%)				
I/II	19 (6)	10 (6)	9 (5)	1
III/IV	321 (94)	167 (93)	154 (94)	
Unknown	3 (1)	2 (1)	1 (1)	
Lymphatic invasion, n (%)				
NO	40 (12)	18 (10)	22 (13)	0.326
Unknown	210 (61)	107 (60)	103 (63)	
YES	93 (27)	54 (30)	39 (24)	
Neoplasm histologic grade, n (%)				
G1/G2	42 (12)	21 (12)	21 (13)	0.67
GX/Unknown	8 (2)	3 (2)	5 (3)	
G3/G4	293 (85)	155 (87)	138 (84)	
Radiotherapy, n (%)				
NO	321 (94)	170 (95)	151 (92)	0.382
YES	22 (6)	9 (5)	13 (8)	
Tumor residual disease, n (%)				
No/Unknown	94 (27)	45 (25)	49 (30)	0.389
YES	249 (73)	134 (75)	115 (70)	
Venous invasion, n (%)				
NO	32 (9)	14 (8)	18 (11)	0.571
Unknown	251 (73)	132 (74)	119 (73)	
YES	60 (17)	33 (18)	27 (16)	

#### A comparison of the CCR5 expression level between the normal tissues and ovarian cancer tissues and the comparison of survival data

**Fig. 1:**
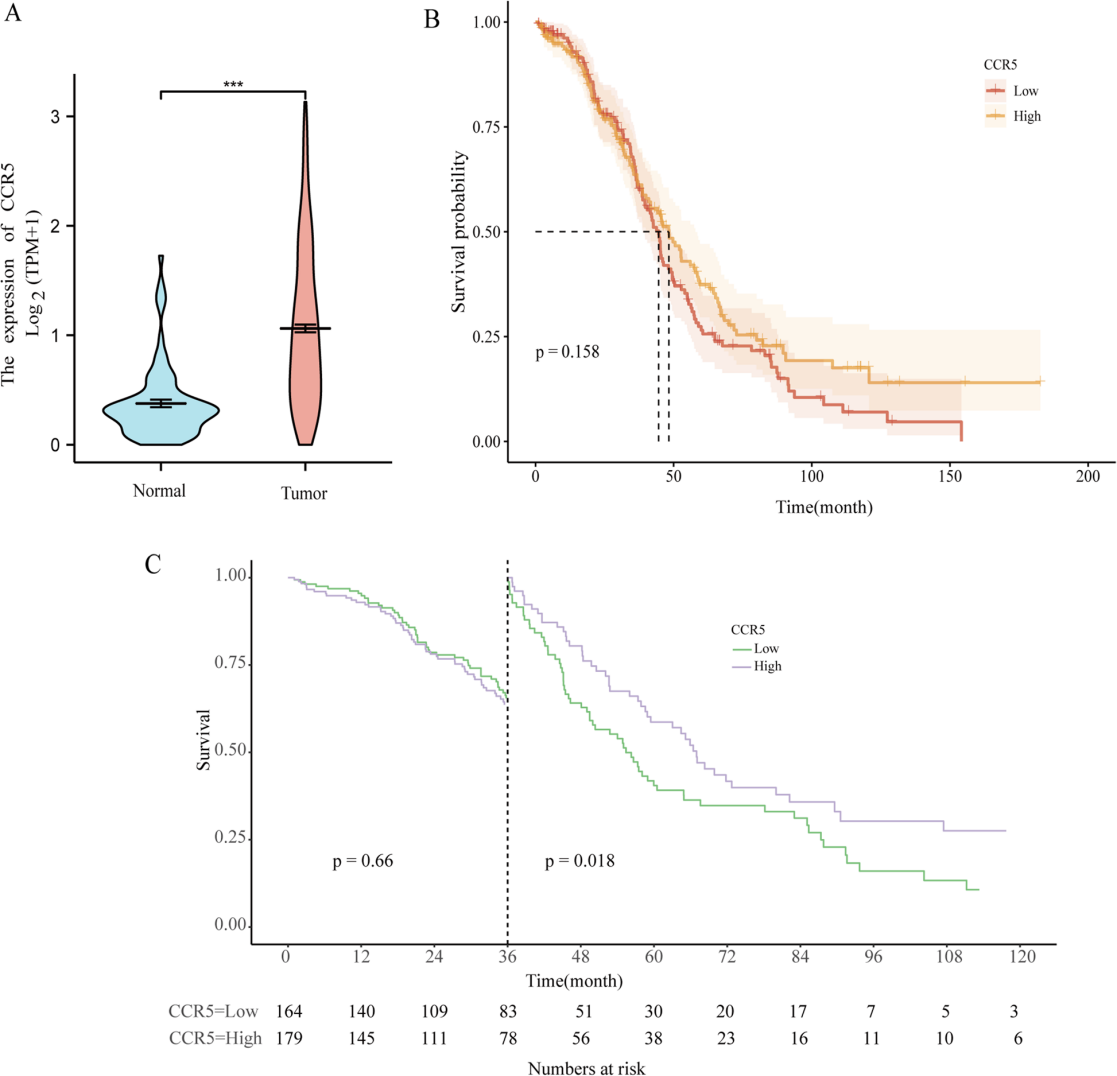
A comparison of the CCR5 expression level between the normal tissues and ovarian cancer tissues and the comparison of survival data. **A** The expression level of CCR5 in OV tissues was significantly higher than that in normal tissues; **B** The Kaplan–Meier curve shows no significant difference in patients’ OS between two groups with different CCR5 expression levels; **C** Landmark analyses at 36 months showed a higher rate of OS for patients with high-expression of CCR5, whereas landmark analysis at 12 and 60 months showed no significant difference between the two groups

The expression level of CCR5 in OV tissues was significantly higher than that in normal tissues(P<0.001), as shown in Fig. 1(A).

Figure 1(B) demonstrates the Kaplan-Meier survival curves of patients in the two groups of different CCR5 expressions. It indicates that patients with low-expression CCR5 were associated with worse OS than patients with high-expression CCR5, with a median OS of 44,53 months vs. 48.27 months. However, the result was not statistically significant (*p*=0.158).

Landmark analysis shows a higher rate of OS for the high expression CCR5 group at a late stage (*p*=0.018) with a 36-month landmark, while no statistical difference was found at an early stage (*p*=0.66) and at the other landmark time (Fig. 1(C))

#### Associations between overall survival and clinicopathological characteristics using Cox regression: CCR5

**Fig. 2:**
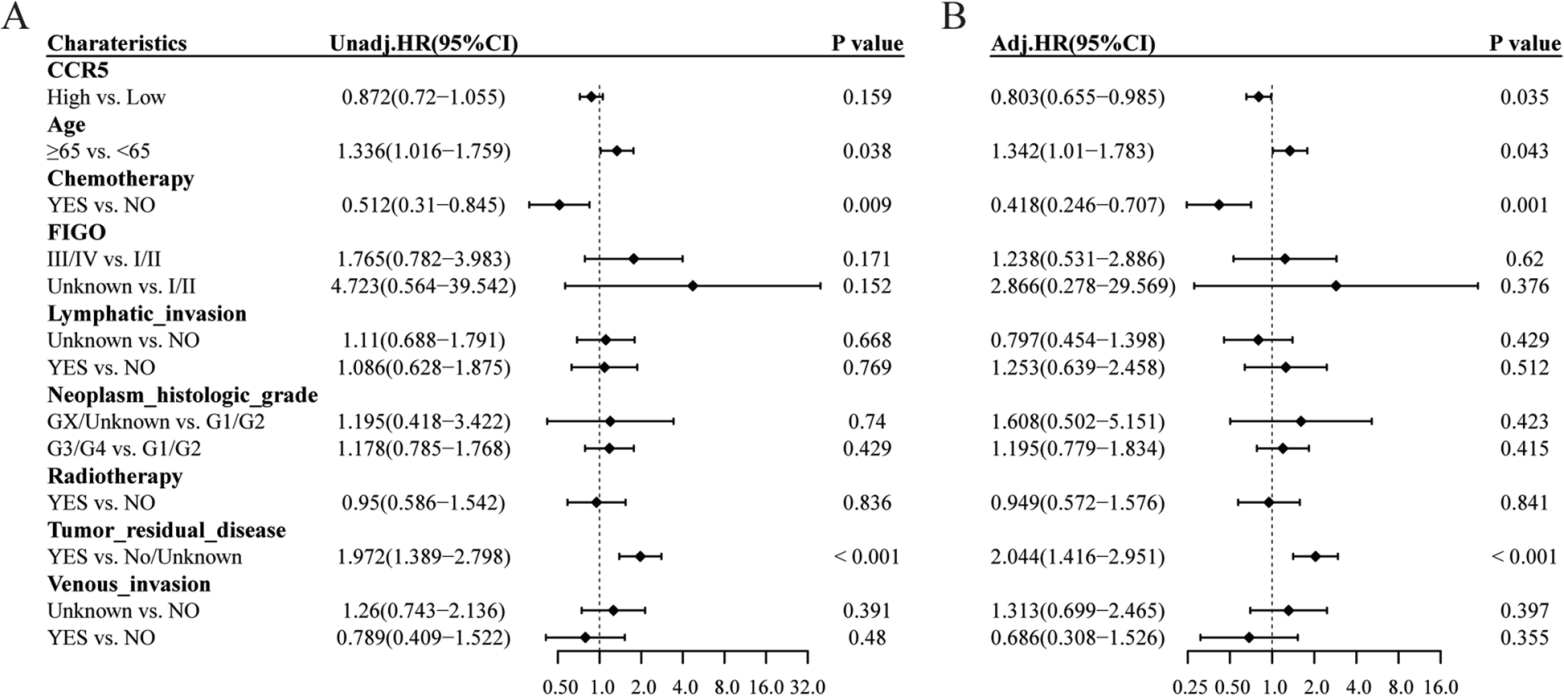
Associations between overall survival and clinicopathological characteristics using Cox regression: CCR5. **A** Univariate Cox regression analysis revealed that high expression of CCR5 was a protective factor for OS, although the result was not statistically significant. **B** With multivariate Cox regression analysis, the result demonstrated a significant correlation between CCR5 expression and OS. High expression of CCR5 remained to be a protective prognostic factor

Univariate Cox regression analysis revealed that high expression of CCR5 was a protective factor for OS although the result was not statistically significant (HR=0.872， 95%CI:0.72-1.055，*p*=0.159) (Fig. 2A). With multivariate Cox regression analysis, however, the result demonstrated a significant correlation between CCR5 expression and OS (HR=0.803， 95%CI: 0.655-0.985，*p*=0.035) High expression of CCR5 remained to be a protective prognostic factor. (Fig. 2B)

### ROC curves for predicting OS of ovarian cancer by CCR5 expression in the survival model

**Fig. 3:**
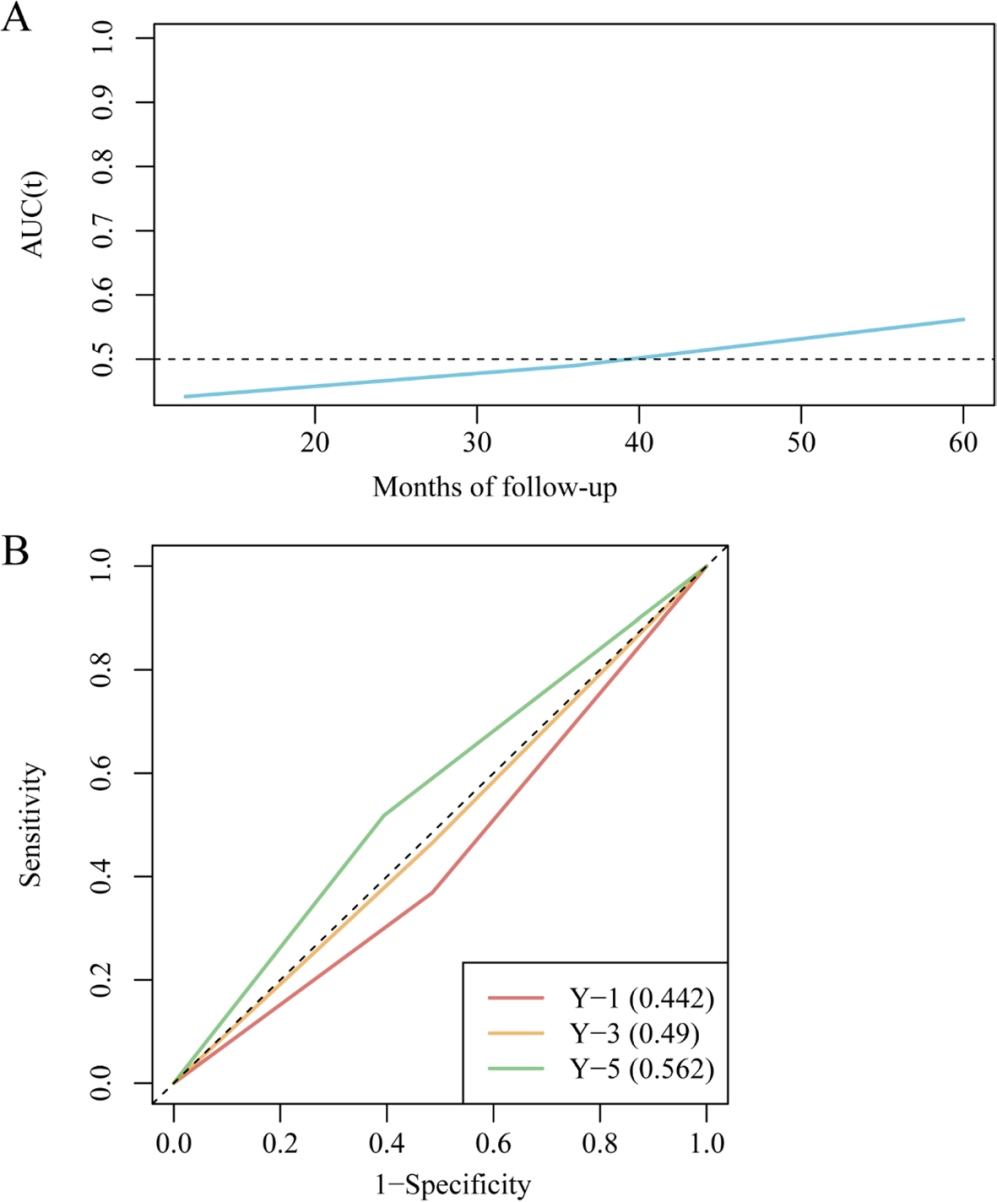
ROC curves for predicting OS of ovarian cancer by CCR5 expression in the survival model (**A**) The time-dependent AUC according to years of follow-up; (**B**) The designated ROC curves at 1, 3, and 5 years of follow-up; the areas under the ROC curves are 0.442, 0.49, and 0.562, respectively

The time-dependent ROC curve was plotted, and the AUC was 0.442, 0.490, 0.562 for 12 months, 36 months, and 60 months, respectively (Fig. 3).

### Relationship between CCR5 expression level and clinicopathological characteristics

**Fig. 4:**
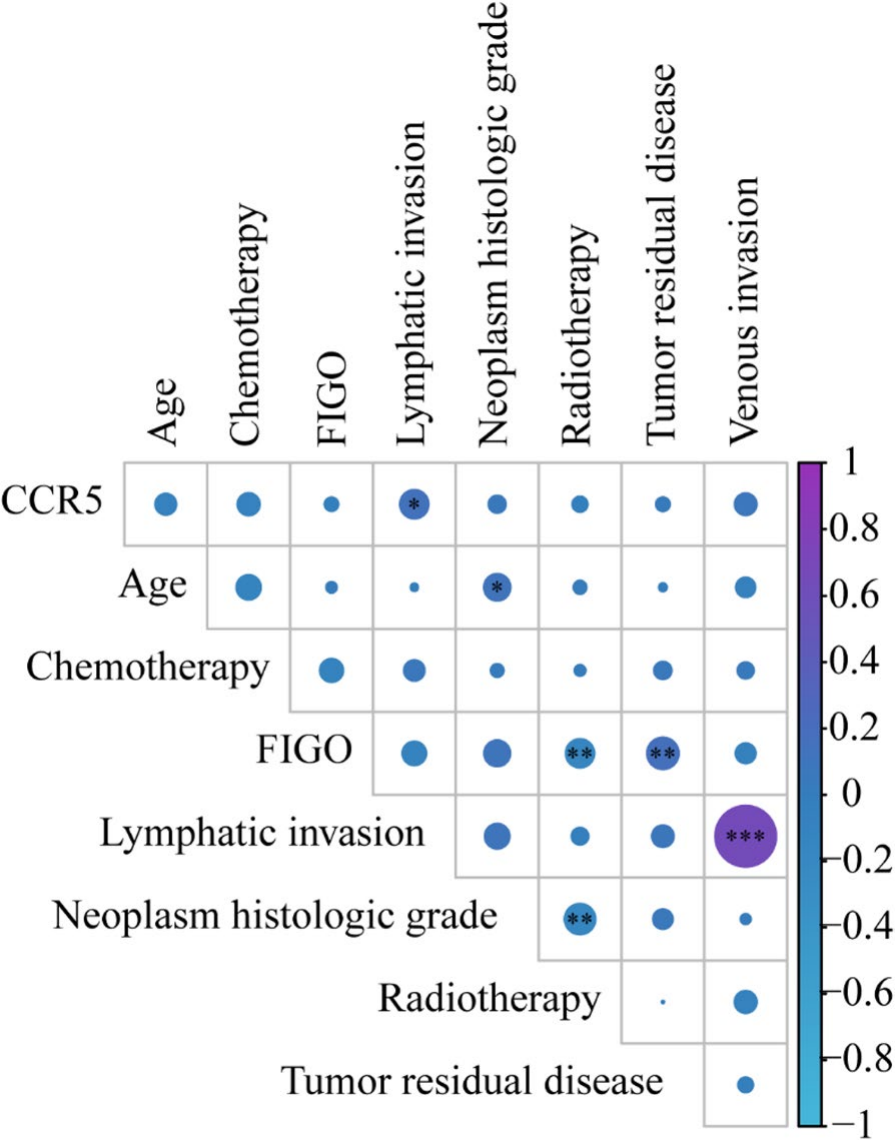
Relationship between CCR5 expression level and clinicopathological characteristics. It was observed that the expression of CCR5 correlated positively with the lymphatic invasion

Specifically, it was observed that the expression of CCR5 was negatively correlated with age, chemotherapy, FIGO stage, and radiotherapy. In contrast, its expression was positively correlated with lymphatic invasion, neoplasm histologic grade, tumor residual disease, and venous invasion, although only its correlation with lymphatic invasion was statistically significant. (*p*<0.05) (Fig. 4)

### Relationship between CCR5 expression level and abundance of immune infiltrates

It was observed in the heat map of Spearman’s rank correlation that the expression level of CCR5 correlated positively with the relative abundance of dendritic cells activated(*p*<0.05). By contrast, CCR5 was negatively related to B cells naive (*p*<0.05); CCR5 was also found not to be significantly correlated with B cells memory. (Supplemental figure 2)

### GO and KEGG enrichment analysis of the CCR5

The most significantly enriched GO categories in the high-expression CCR5 group compared to the others were for neuromodulation and protease metabolism process **(**Supplemental figure 3**)**. The results of KEGG analysis showed that pathways in the cell cycle, tumor necrosis factors, and m-TOR signaling pathway were mainly enriched.

### Radiomics feature extraction and model establishment

#### Consistency evaluation, Feature Selection, and Construction of the radiomics model

The interobserver ICC was ≥ 0.75, 0.51–0.74, < 0.5 for 95(88.8%), 9(8.4%) and3(2.8%) of the features, respectively. The median ICC was 0.94.

Four optimal features, called original_glcm_Idn, original_gldm_GrayLevelNonUniformity, original_glrlm_RunEntropy, and original_shape_MinorAxisLength (The overall importance was 1.271, 0.445, 0.763 and 0.426, respectively) were selected by the LASSO algorithm among a total of 95 features after ICC analysis. (Supplemental figure 4) There were statistical significances in these four features between the high-grade group and the low-grade group of CCR5 (*p*<0.05). (Supplemental figure 5), (Supplemental Table 2)

We construct the radiomics signature with a Radscore calculated using the following formulas:

$$\mathrm{Rad}-\mathrm{score}=0.409-0.603\ast(\mathrm{original}\;\mathrm{glcm}\;\mathrm{Idn})-0.236\ast(\mathrm{original}\;\mathrm{gldm}\;\mathrm{GrayLevelNonUniformity})-0.301\ast(\mathrm{original}\;\mathrm{glrlm}\;\mathrm{RunEntropy})-0.227\ast(\mathrm{original}\;\mathrm{shape}\;\mathrm{MinorAxisLength})$$

Features’ importance was shown in Supplemental figure 6.

#### The performance of the radiomics model for predicting the CCR5 expression level with 10-fold cross-validation

**Fig. 5:**
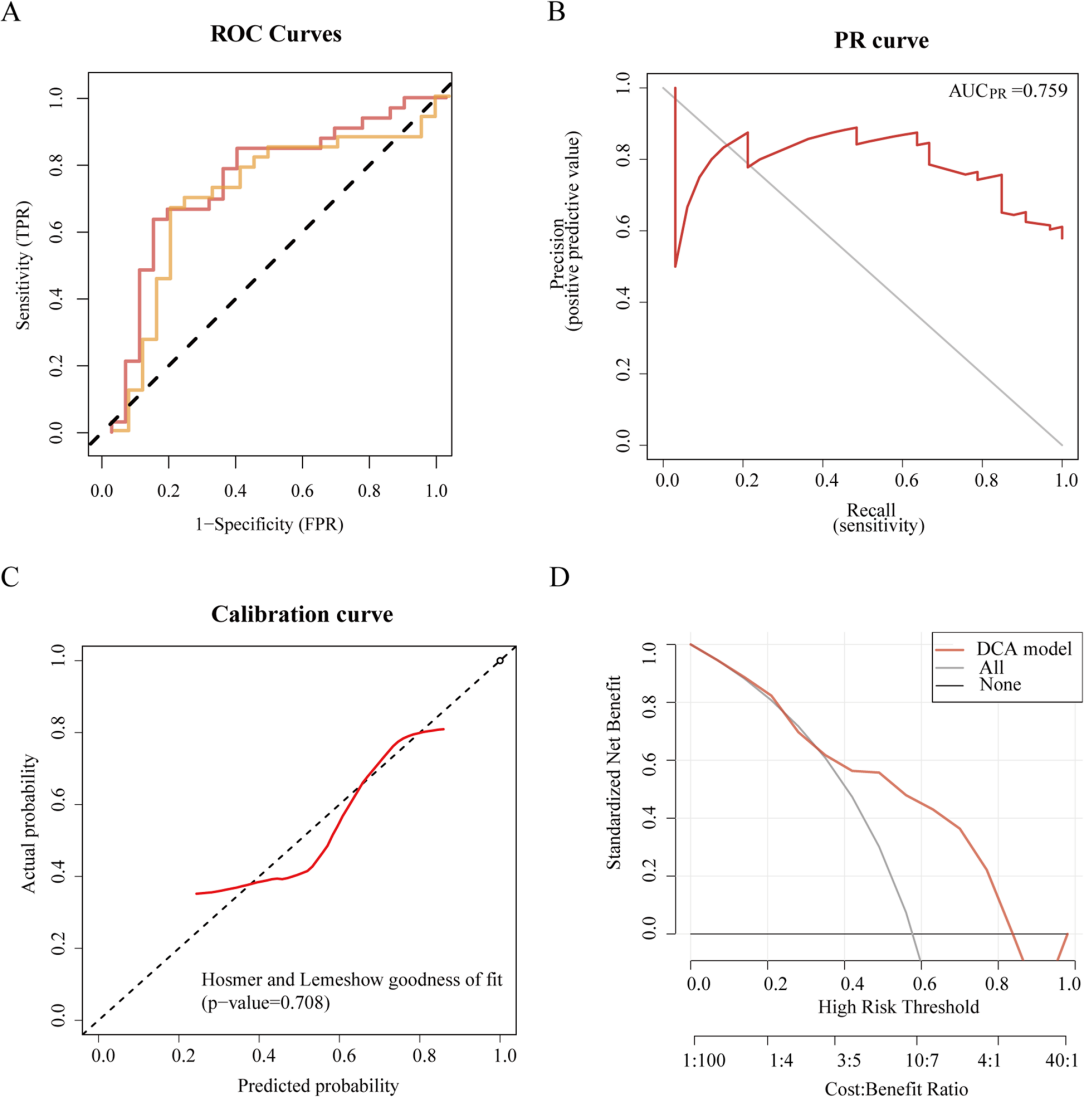
The performance of the radiomics model for predicting the CCR5 expression level with 10-fold cross-validation on the training and validation set. **A** Receiver operating characteristic (ROC) curves of the model. **B** Precision-Recall (PR) curve of the model. The X-axis of the recall curve is the actual positive rate (Recall), and the Y-axis is the precision rate. The area under the curve (AUC)-PR is the average accuracy calculated for each coverage threshold. The more convex the turn to the upper right, the better the model's performance. **C** Calibration curves of the model. A calibration curve describes the consistency between the predicted and gene expression levels. The 45-degree dotted black line represents the ideal prediction performance; the solid red line represents the model's prediction performance. The closer the solid red line is to the ideal dotted line, the better the model's prediction accuracy. **D** Decision curve analysis (DCA) for the model. The y-axis measures the net benefit. The red curve represents the radiomics model; the gray curve represents the assumption that all patients were treated and the straight black line at the bottom of the figure represents the assumption that no patients were treated

In the training set, the ACC, SPE, SEN, PPV and NPV of the radiomics model was score 0.737, 0.875, 0.636, 0.875, 0.636 respectively, with a ROC-AUC of 0.770, and a brier score of 0.19.In the validation set, he ACC, SPE, SEN,PPV and NPV of the radiomics model was score 0.737, 0.833, 0.667, 0.846, 0.645 respectively, with a ROC-AUC of 0.726, and a brier score of 0.212. The PR-AUC of the model was 0.759 and DCA demonstrated its preferable clinical practicality (Fig. 5)

#### Difference between radiomics score when compared using Wilcoxon test in the training and validation set

The radiomics score (RS) was significantly higher in high-expression CCR5 group than in low-expression CCR5 group both in training and validation set (*p*<0.05). (Supplemental figure 7)

### Model evaluation and clinical application

#### Association between Radiomics score and patients’ OS

**Fig. 6:**
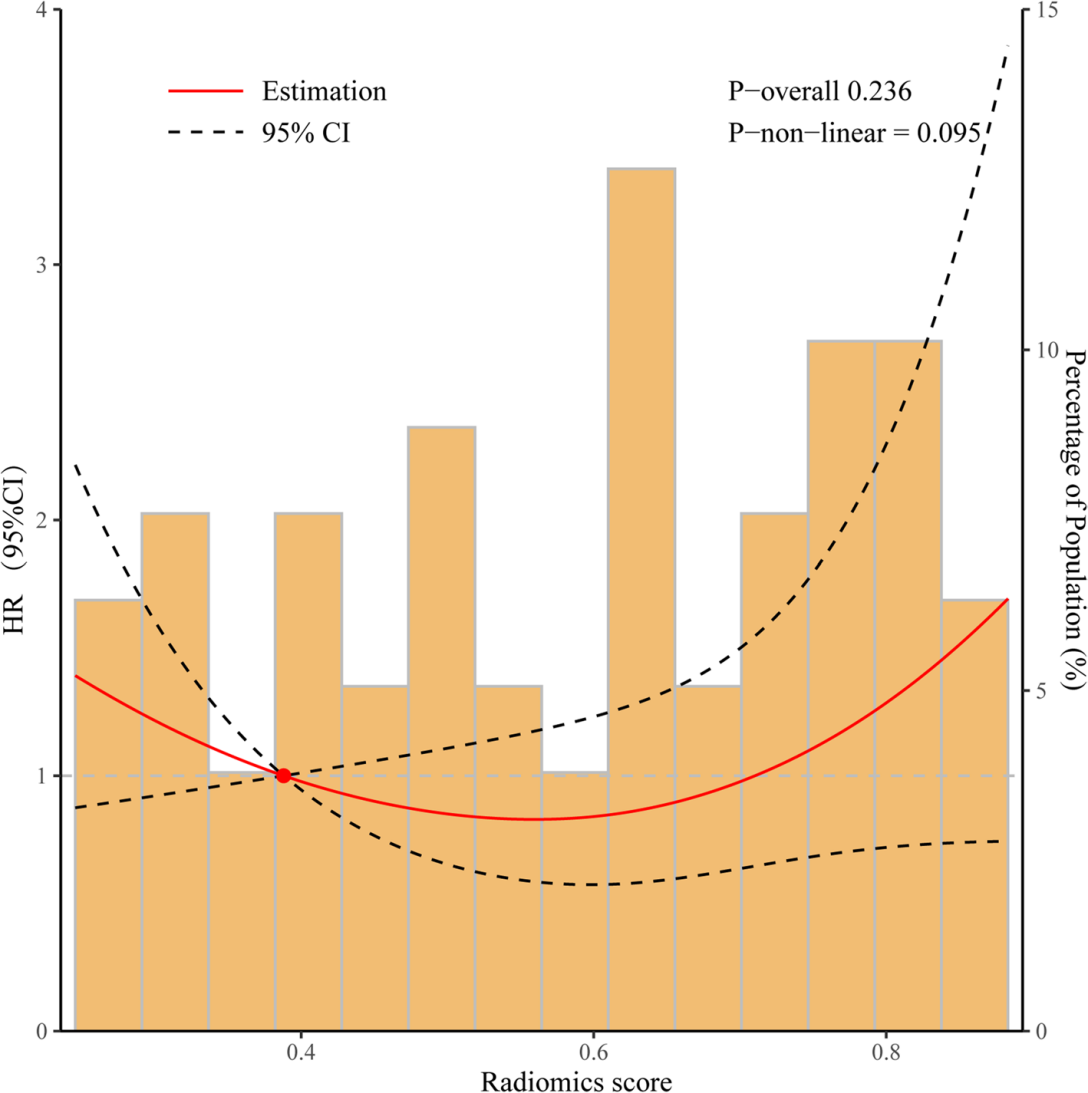
Association between Radiomics score and patients’ OS. The curve of HR versus RS using restricted cubic splines. The solid red line indicates HR, and 95% CI is denoted by the dotted black lines

Figure 6 shows when we choose the cutoff of RS (=0.388) as reference, it exists a linear dose-response relationship between RS and overall survival of patient with ovarian cancer as analyzed by RCS (*p* overall≥0.05, *p*-non-linearity≥0.05).

#### Clinical characteristics of the population with high and low RS group

Comparisons of baseline characteristics are shown in Table 2. No statistical difference was found in age, chemotherapy, FIGO stage, lymphatic invasion, neoplasm histologic grade, radiotherapy, tumor residual disease and venous invasion between the high (*n*=70) and low (*n*=19) RS group.

**Table 2:** Clinical characteristics of the population with high and low Radiomics score groups

Variables	Total (n = 89)	Low (n = 19)	High (n = 70)	p
Age, n (%)				
<65	57 (64)	9 (47)	48 (69)	0.15
≥65	32 (36)	10 (53)	22 (31)	
Chemotherapy, n (%)				
NO	4 (4)	2 (11)	2 (3)	0.199
YES	85 (96)	17 (89)	68 (97)	
FIGO, n (%)				
II/III	47 (53)	8 (42)	39 (56)	0.427
IV/Unknown	42 (47)	11 (58)	31 (44)	
Lymphatic invasion, n (%)				
NO/Unknown	66 (74)	13 (68)	53 (76)	0.56
YES	23 (26)	6 (32)	17 (24)	
Neoplasm histologic grade, n (%)				
G1/G2	9 (10)	2 (11)	7 (10)	1
G3/GX	80 (90)	17 (89)	63 (90)	
Radiotherapy, n (%)				
NO	86 (97)	18 (95)	68 (97)	0.518
YES	3 (3)	1 (5)	2 (3)	
Tumor residual disease, n (%)				
No/Unknown	32 (36)	7 (37)	25 (36)	1
YES	57 (64)	12 (63)	45 (64)	
Venous invasion, n (%)				
NO/Unknown	73 (82)	17 (89)	56 (80)	0.506
YES	16 (18)	2 (11)	14 (20)	

#### The comparison of survival data

**Fig. 7:**
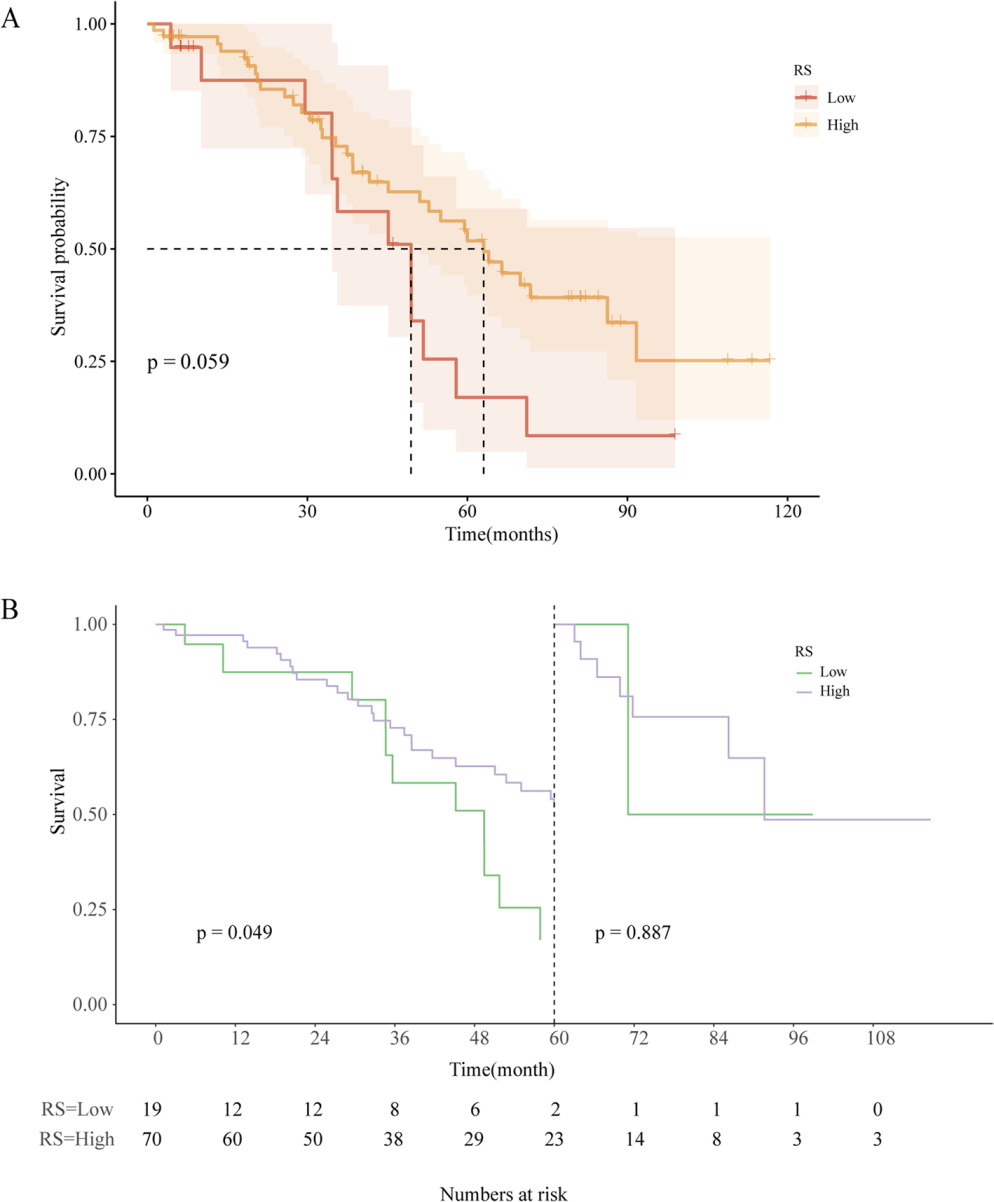
The comparison of survival data (**A**) The Kaplan–Meier curve shows no significant difference in patients’ overall survival (OS) between two groups with different radiomics scores (RS); (**B**) Landmark analyses at 60 months showed a higher rate of OS for patients with high RS, whereas landmark analysis at 12 and 36 months showed no significant difference between the two groups

Figure 7(A) demonstrates the Kaplan-Meier survival curves of patients in the two groups. It shows that patients with low RS were associated with worse OS than patients with high RS, with a median OS of 49.43 months vs. 63.03 months. However, the result was not statistically significant (*P*=0.059). Landmark analysis (Fig. 7(B))shows a higher rate of OS for high RS group at an early stage (*p*=0.049) with a 60-month landmark while no statistical difference was found at late stage (*p*=0.887) and at the other landmark time.

#### Nomogram and model evaluation

**Fig. 8:**
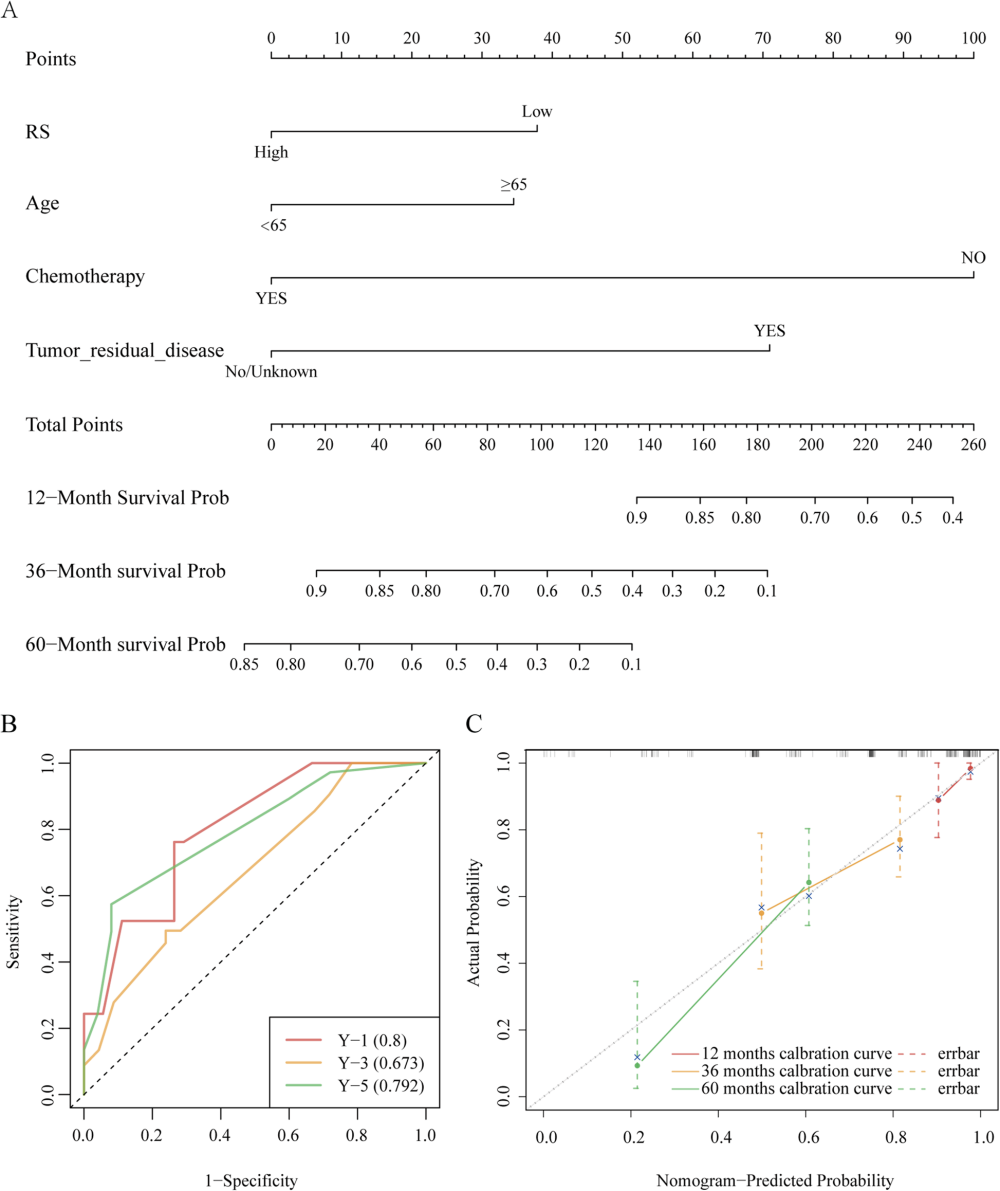
Nomogram and model evaluation (**A**) Creation of the nomogram to predict the overall survival of a patient with ovarian cancer. **B** The time-dependent ROC of the risk score (**C**) Calibration curves of the risk score

Besides RS, clinical characteristics including age, chemotherapy, tumor residual diseases were contained in the model using stepwise logistic regression with minimum AIC method. A predictive nomogram was generated based on the total score of each patient. For each person, every selected variable pointed to a score according to the above scale, and we could get a total score by summing up all scores (Fig. 8(A)). The AUC of the risk score of the time-dependent ROC was 0.8, 0.673 and 0.792 for 1-year, 3-year and 5-year, respectively (Fig. 8(B)). Compared to every single predictor, the predictive nomogram achieved the best performance with the highest ROC-AUC (Supplemental table 3). It appeared that all three calibration curves of 1-year, 3-year and 5-year were closed to the standard curve. (Fig. 8(C))

## Discussion

In this integrated radiomics-molecular analysis based on CT images, we aimed to explore the correlations between the pre-treatment radiomics profile of ovarian cancer and CCR5 expression levels and overall survival. Our results demonstrated that CT features are associated with differences in CCR5 expression levels and overall survival of ovarian cancer, indicating that additional molecular information can be obtained from radiomics analysis of CT images which might provide a new method for therapeutic decision-making.

Due to the heterogeneity of ovarian cancers, the survival rate of patients varies widely. 15% of women with ovarian cancer die within 2 months of diagnosis, while almost half are expected to survive beyond five years [37]. Accurate prediction of prognosis is pivotal for the management of ovarian cancer. CCR5 as a chemokines’ receptor, which may promote invasion and metastasis [6], has been actively investigated in various cancers [7–13]. Although limited in the field of ovarian cancer, several studies reported that CCR5 might be associated with prognosis [19, 20]. Recently, the successful applications of radiomics in ovarian cancer, including cancer detection, differentiation diagnosis, prediction of treatment response, survival and lymph node metastasis [21, 28–33], suggested that radiomics may hold potential for decoding the CCR5 status in patients with ovarian cancer. Therefore, we conducted the present study. Landmark analyses at 60 months showed a higher rate of OS for patients with high radiomics scores, which demonstrated that higher expression levels of CCR5 are linked to better prognosis in patients with ovarian cancer (*p* < 0.05). We believe that noninvasive predicting the expression levels of CCR5 based on radiomics is helpful for nuanced clinical judgments.

Recently, a wealth of critical molecular markers, other prediction information, and the development of molecular biological and artificial intelligence techniques owed cancer researchers. Wingfield [5] applied radiomics features to predict the expression levels of CD44 and CD133 in lower-grade gliomas; In the training and validation sets, the model yielded AUCs of 0.912 and 0.805, respectively, in the CD44 model; 0.912 and 0.816, respectively, in the CD133 model. Xiao [38] applied a radiomics model to predict SYP gene expression; The prediction model yielded an accuracy of 0.93. In our study, after features screening based on repeat LASSO using the optimal lambda of -2.349, four optimal features: original_glcm_Idn, original_gldm_GrayLevelNonUniformity, original_glrlm_RunEntropy, and original_shape_MinorAxisLength were selected for constructing the model. The predictive performance of models predicting CCR expression levels was good in the training and validation sets, and yielded AUCs of 0.770 and 0.726, respectively. In our research, restricted cubic spline models were used to fit the relationship between RS and OS and identify the optimal cut-off value of RS. It showed good predictive power with the time-dependent 0.679, 0.552, and 0.613 for 1-year, 3-year, and 5-year survival using single RS as predictor and AUROC of 0.8, 0.673, and 0.792 using RS combined with clinical features. The results demonstrated that combining clinical and radiomics models improved model performance. This finding was consistent with the research of Avesani [39], who built predictive radiomics models for early relapse and BRCA mutation based on a multicentric database of OC. Their models showed low performance in predicting both BRCA mutation and 1-year relapse with traditional radiomics (AUC: 0.46-0.59 for BRCA and 0.46-0.56 for relapse) and deep learning (AUC of 0.48 BRCA and 0.50 for relapse). The inclusion of clinical variables improved the performance of the radiomics models to predict BRCA mutation (AUC in the test set of 0.74).

Accurate survival prediction is important for decision making, especially for patients with cancers.

Decision-making, as one of the core tenets of medicine, relies upon integrating quantitative data. Many indicators were used to predict the survival of OC. Zhang et al. used indicator CA125 combined with D-dimer (ICD) in predicting OS in patients with OC. They showed that ICD (HR 2.651, 95% CI 1.273–5.520, *p*=0.009) was an independent predictor of OS in ovarian cancer, although the predictive power was limited [40]. Qi constructed a predictive nomogram based on risk factors for OS, including age, laterality, and American Joint Committee on Cancer (AJCC) stage. The C-index of the OS nomograms were 0.85 (95% CI: 0.81-0.89) and 0.80 (95% CI: 0.74-0.87), respectively in the training and validation cohort [41]. These predictors usually require the invasive operation or experienced staff, being labor-intensive, time-consuming, and possibly leading to interobserver bias, while the predictive power was not ideal. In the last few years, more and more new techniques have begun paving the way towards personalized and precision medicine based on the increasing knowledge of the tumoral microenvironment at a molecular level. Radiomics represents a recently introduced translational field of study, aiming to find relationships between multilayered information extracted from imaging examinations and clinical data to support evidence-based clinical judgement [42]. Imaging biomarkers could be used as predictors of OS. Several previous papers have reported a significant correlation between radiomics based on CT images and survival in ovarian cancer patients (summarized in supplemental Table 4) and have shown the good performance of the prediction model [43–49]. These results are consistent with those of our analysis. This consistency suggests that the radiomics offer good prediction power in ovarian cancer. In this work, we built prognostic models based on a biomarker, radiomics, and clinical features for survival analysis of patients with ovarian cancer. The model was proved to be a significant predictor of overall survival. We provided a noninvasive tool with relatively high accuracy to predict the OS of ovarian cancer using the CT-based machine learning radiomics predicting CCR5 expression level. Our findings suggested that combining the biomarker-based features into the standard radiomics might offer a useful approach for improving the prognostic prediction accuracy with a view to clinical use.

Some limitation of our research needs to be considered. Firstly, all image information was acquired from public dataset TCIA, which inevitably contain variance in the quality of images that may have an impact on predictive analysis, Secondly, this study is a retrospective study with a relatively small sample size, so the generalizability still has to be studied. Thirdly, in our study, ROI delineating each tumor was manually drawn under the supervision of two experienced radiologists, while manual delineation is a critical task, especially because it carries a certain amount of subjectivity. As reported in a recent study [50], although with high accuracy, manual segmentation by radiologists is labor-intensive, time-consuming, and not always feasible for radiomics analysis requiring huge datasets. Additionally, manual segmentation is subject to inter- and intra-observer variability [51]. Hence, many semi-automatic delineation algorithms are applied in the clinical practice although less precise than manual segmentation [50]. On the other hand, in the case of manual delineations performed by different radiologists, one solution is the STAPLE tool [52] that can be used to overcome the limitation by producing a consolidated reference between the different operators. So, to reduce the operator interaction in the segmentation process and to improve the reproducibility of radiomics studies, automatic or semi-automatic approaches should be used in further study.

## Conclusion

In conclusion, the expression levels of CCR5 can significantly influence the prognosis of patients with ovarian cancer. We developed a model from radiomics can effectively predict the expression levels of CCR5. Our model therefore has the potential to be widely used as a practical tool for the noninvasive characterization of tumor.

## Supplementary Information

**Additional file 1: Supplemental Table 1.** Inclusion/exclusion criteria applied to determine the study samples. **Supplemental Table 2.** Formula of the Radiomics model. **Supplemental Table 3.** AUC of the time-dependent ROC with different predictor. **Supplemental Table 4.** The image processing and feature extraction. **Supplemental Figure 1.** The image processing and feature extraction. **Supplemental Figure 2.** Relationship between CCR5 expression level and abundance of immune infiltrates in the study cohort. **Supplemental Figure 3.** GO and KEGG enrichment analysis of the CCR5. **Supplemental Figure 4.** Feature Selection of the radiomic model. **Supplemental Figure 5.** Comparison of the four optimal radiomic features between high-expression and low-expression CCR5 group. **Supplemental Figure 6.** Radiomic features' importance according to Logistic regression. **Supplemental Figure 7.**  Difference between radiomics score when compared using Wilcoxon test in the training (A)and validation (B) set. **Supplemental Figure 8.**  Principal Component Analysis (PCA) performed on the extracted features to plot data in a space of reduced dimensions.
