# Supplementary material for: CT-based machine learning radiomics predicts CCR5 expression level and survival in ovarian cancer
Source: J Ovarian Res. 2023 Jan 3;16:1. doi: 10.1186/s13048-022-01089-8 (PMC9809527; doi:10.1186/s13048-022-01089-8)

**Supplemental Table1**: Inclusion/exclusion criteria applied to determine the study samples

| **TCGA** | Excluded samples | Remaining samples |
| --- | --- | --- |
| Total samples | / | 379 |
| Screening: primary ovarian cancer | 25 | 354 |
| Excluded: OS=NA | 1 | 353 |
| Excluded: OS＜30 | 10 | 343 |
| **TCIA-CT** |  |  |
| Total samples | / | 143 |
| Excluded image：post-operation, poor quality | 52 | 91 |
| Intersection of TCGA genomic data | 34 | 57 |
| **TCGA-OV clinical data** |  |  |
| Total samples | / | 600 |
| Excluded: OS/OS.time=NA | 18 | 582 |
| Excluded: OS.time＜30 | 12 | 570 |
| Intersection of TCIA clinical data | 481 | 89 |

CT: computed tomography; OS: overall survival; OV: ovarian cancer; TCIA: Cancer Imaging Archive; TCGA: The Cancer Genome Atlas

**Supplemental Table2:** Formula of the Radiomics model

|  | Estimate | Std. Error | z value | Pr(>\|z\|) |
| --- | --- | --- | --- | --- |
| (Intercept) | 0.409 | 0.308 | 1.328 | 0.184 |
| original_glcm_Idn | -0.603 | 0.474 | -1.271 | 0.204 |
| original_gldm_GrayLevelNonUniformity | -0.236 | 0.531 | -0.445 | 0.656 |
| original_glrlm_RunEntropy | -0.301 | 0.395 | -0.763 | 0.446 |
| original_shape_MinorAxisLength | -0.227 | 0.532 | -0.426 | 0.670 |

**Supplemental Table 3**: AUC of the time-dependent ROC with different predictor.

| Predictor | AUC of the time-dependent ROC | | |
| --- | --- | --- | --- |
|  | 1-year | 3-year | 5-year |
| RS | 0.679 | 0.552 | 0.613 |
| Age | 0.441 | 0.552 | 0.689 |
| Chemotherapy | 0.734 | 0.563 | 0.534 |
| FIGO | 0.624 | 0.555 | 0.488 |
| Lymphatic invasion | 0.662 | 0.57 | 0.583 |
| Neoplasm histologic grade | 0.549 | 0.495 | 0.554 |
| Radiotherapy | 0.521 | 0.488 | 0.507 |
| Tumor residual disease | 0.562 | 0.594 | 0.605 |
| Venous invasion | 0.536 | 0.57 | 0.542 |
| Step AIC | 0.800 | 0.673 | 0.792 |

RS: radiomic score; Step AIC: multi-variate stepwise logistic regression with minimum AIC (Akaike Information Criterion) method.

**Supplemental table4:** **The image processing and feature extraction**

| Reference | Publication year and Type | Patient(N) | Feature Included (N) | Main finding |
| --- | --- | --- | --- | --- |
| Lu, H., et al. [44] | 2019, Retrospective | 364 | 4 | Association between 4 features (RPV) and OS. RPV improved the clinical prognostic methods；Association between RPV and PFS. |
| Meier, A., et al. [45] | 2019, Retrospective | 88 | 3 | Association between SE and OS； Association between SCV and SCP with PFS；Association between SE, SCV, SCP and surgical resection status. |
| Zargari, A., et al.[46] | 2018, Retrospective | 120 | 11 | Association between 11 features and PFS. Greater weights for the shape and density features |
| Wei, W., et al. [47] | 2019, Retrospective | 142 | 4 | 4 features associated with prediction of 3-year recurrence. Better performance of the radiomic model than the clinical prognostic model |
| Rizzo, S., et al.[48] | 2018, Retrospective | 101 | 3 | Association between 3 features and 12-months recurrence. The clinical-radiomics model outperformed the clinical model |
| Vargas, H.A., et al. [49] | 2017, Retrospective | 38 | 3 | Association between SE, SCS and SCP and OS ；Association between heterogeneity and surgical resection status |

OS: Overall Survival; PFS: Progression Free Survival; RPV: Radiomic Prognostic Vector; SE: Inter-site Entropy; SCV: Inter-site Cluster Variance; SCP: Inter-site Cluster Prominence; SCS: Inter-site Cluster Shade;

**Supplemental figure1:** **The image processing and feature extraction**


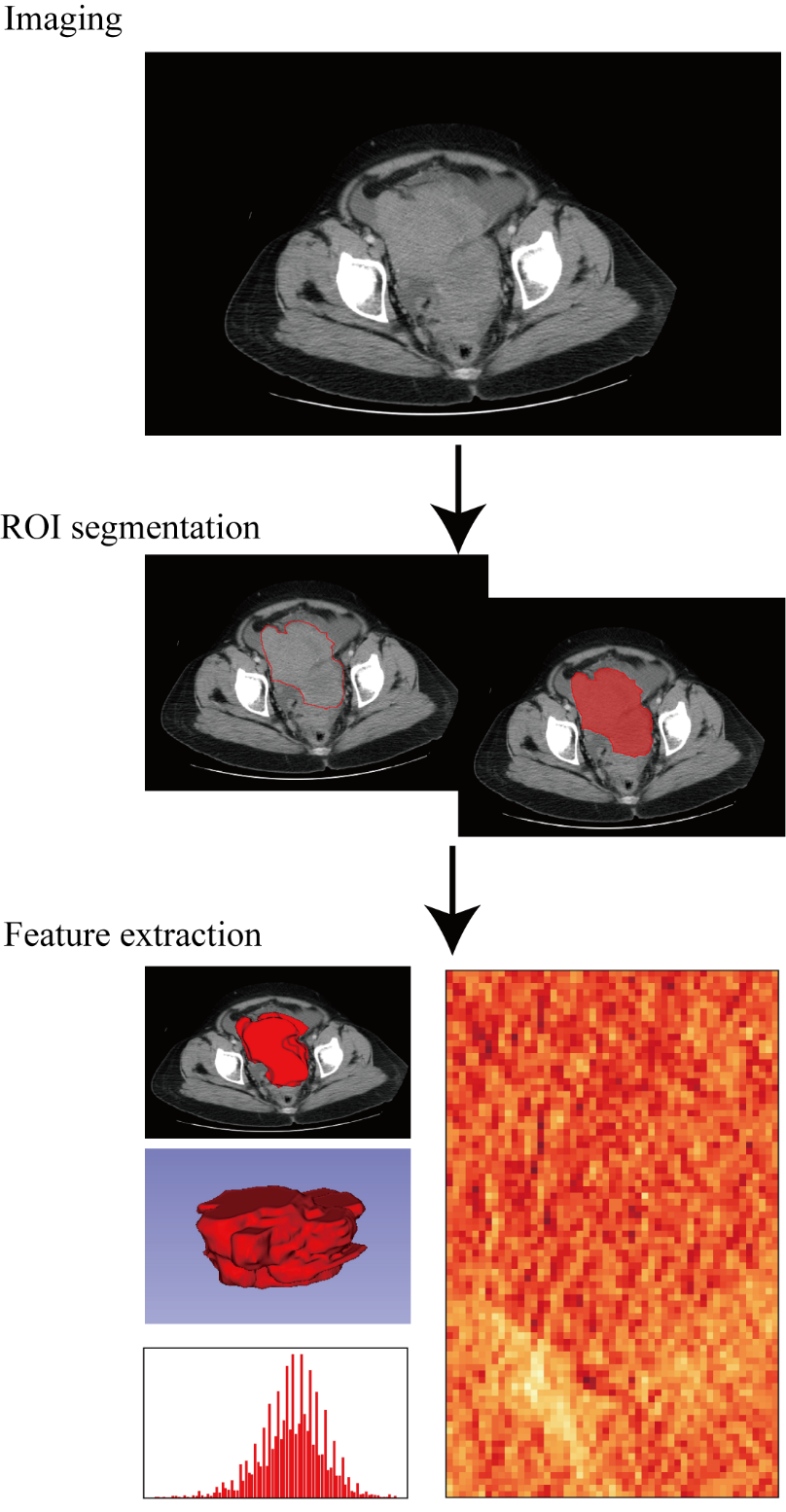


**Supplemental Figure 2. Relationship between CCR5 expression level and abundance of immune infiltrates in the study cohort.** It was observed in the heat map of the Spearman’s rank correlation that the expression level of CCR5 correlated positively with the relative abundance of dendritic cell activated（*p*<0.05）. By contrast, CCR5 was observed negatively related to B cells naive（*p*<0.05）; CCR5 was also found not significantly correlated with B cells memory.


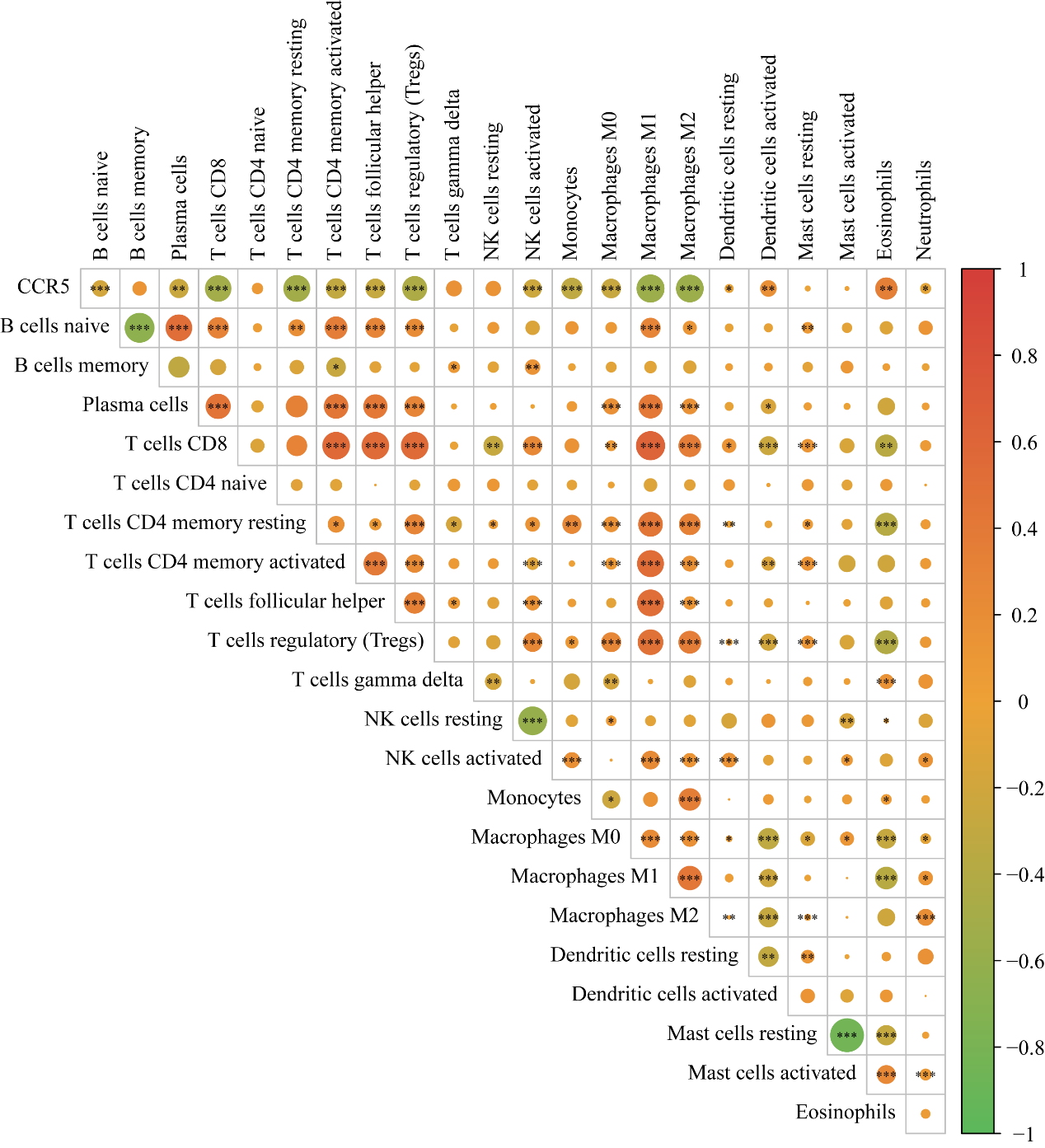


**Supplemental Figure 3: GO and KEGG enrichment analysis of the CCR5**. The most significantly enriched GO categories in high-expression CCR5 group compared to the other were for neuromodulation and protease metabolism process. The results of KEGG analysis showed that pathways in cell cycle, tumor necrosis factors and m-TOR signaling pathway were mainly enriched.


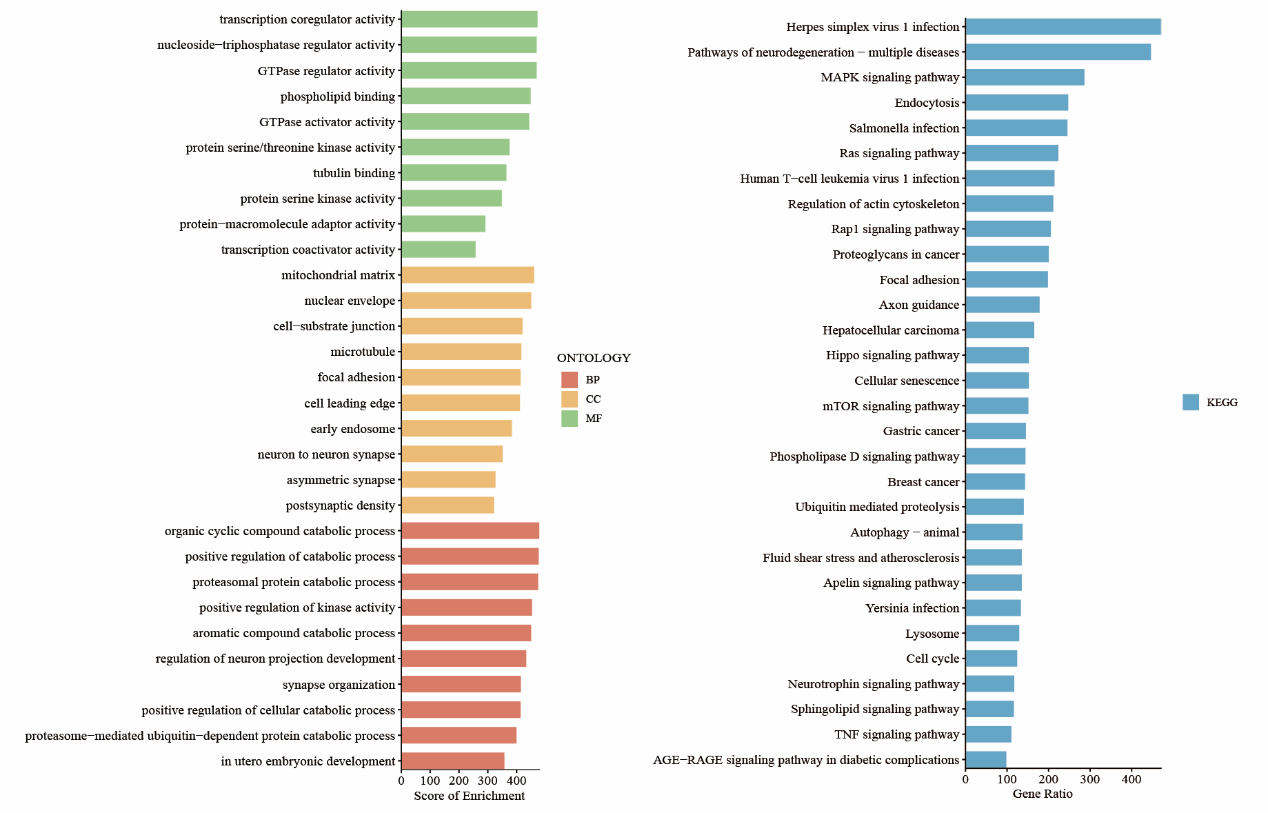


**Supplemental Figure 4：Feature Selection of the radiomic model.** (a) Plot of the ten-fold cross-validation for identification of the optimal lambda (tuning parameter) based on minimizing the partial likelihood deviance error for our image features. (b) Plot of non-zero coefficients or the selected image features in the LASSO logistic regression model by using the optimal lambda of -2.349 (c)Four optimal features: original_glcm_Idn, original_gldm_GrayLevelNonUniformity, original_glrlm_RunEntropy and original_shape_MinorAxisLength were selected from a total 95 features.
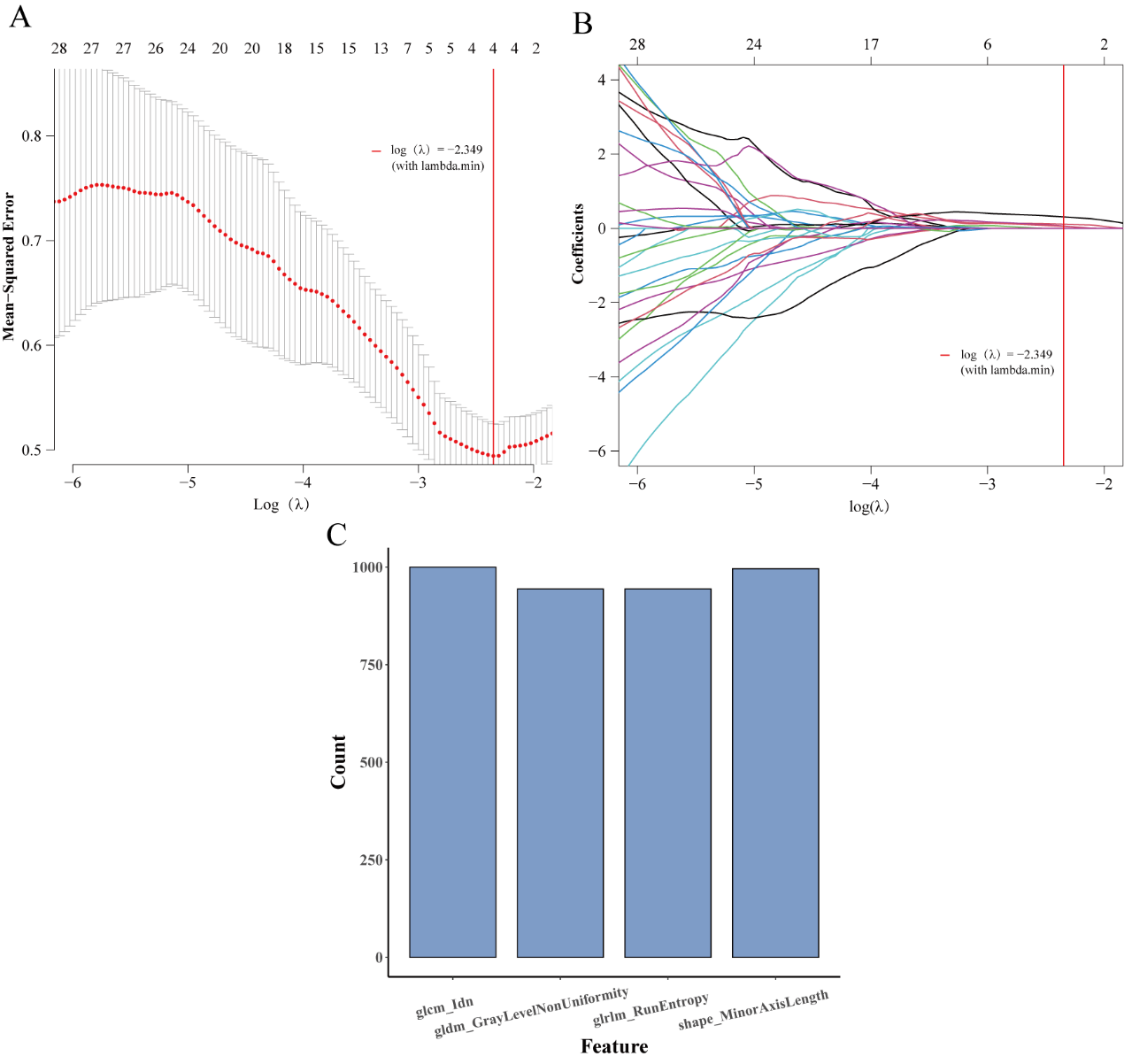


**Supplemental Figure 5: Comparison of the four optimal radiomic features between high-expression and low-expression CCR5 group.**
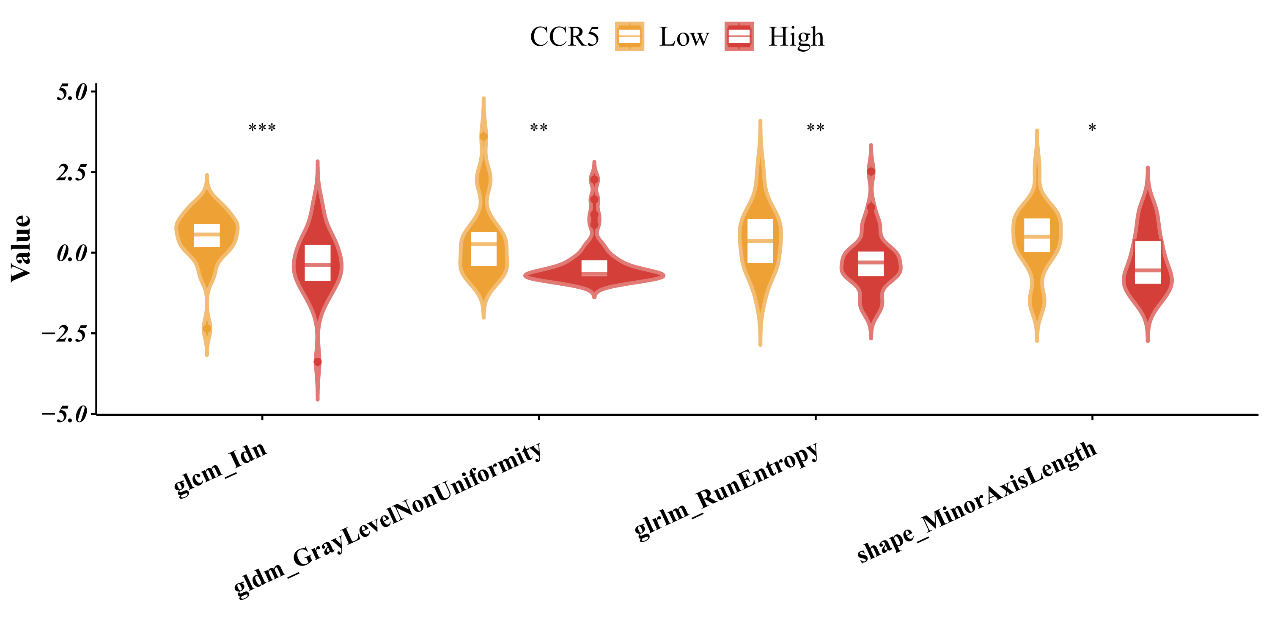


**Supplemental Figure 6: Radiomic features' importance according to Logistic regression.**
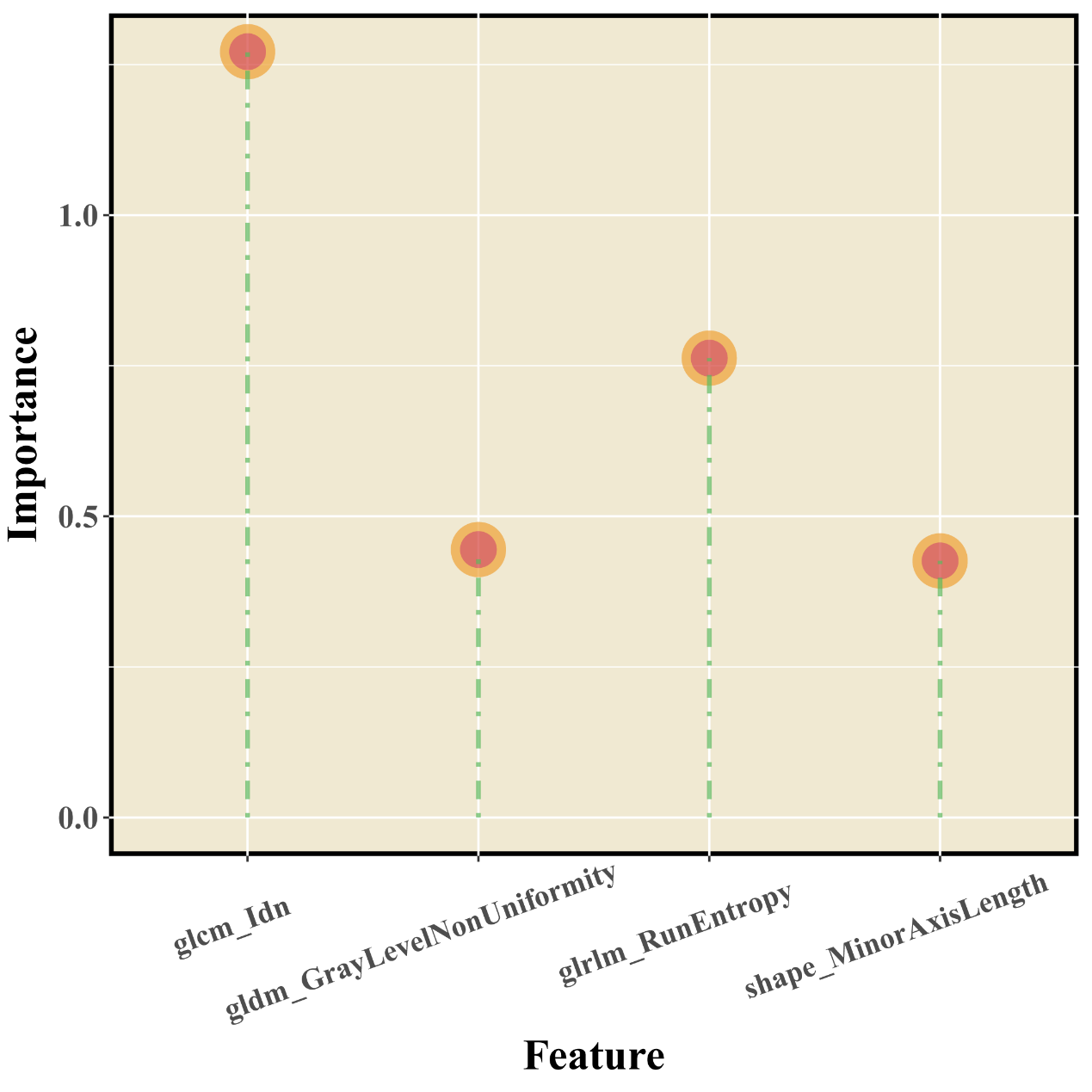


**Supplemental Figure 7: Difference between radiomics score when compared using**

**Wilcoxon test in the training (A)and validation (B) set.** Significant differences were observed between patients with high-expression CCR5 and Low-expression CCR5 group.


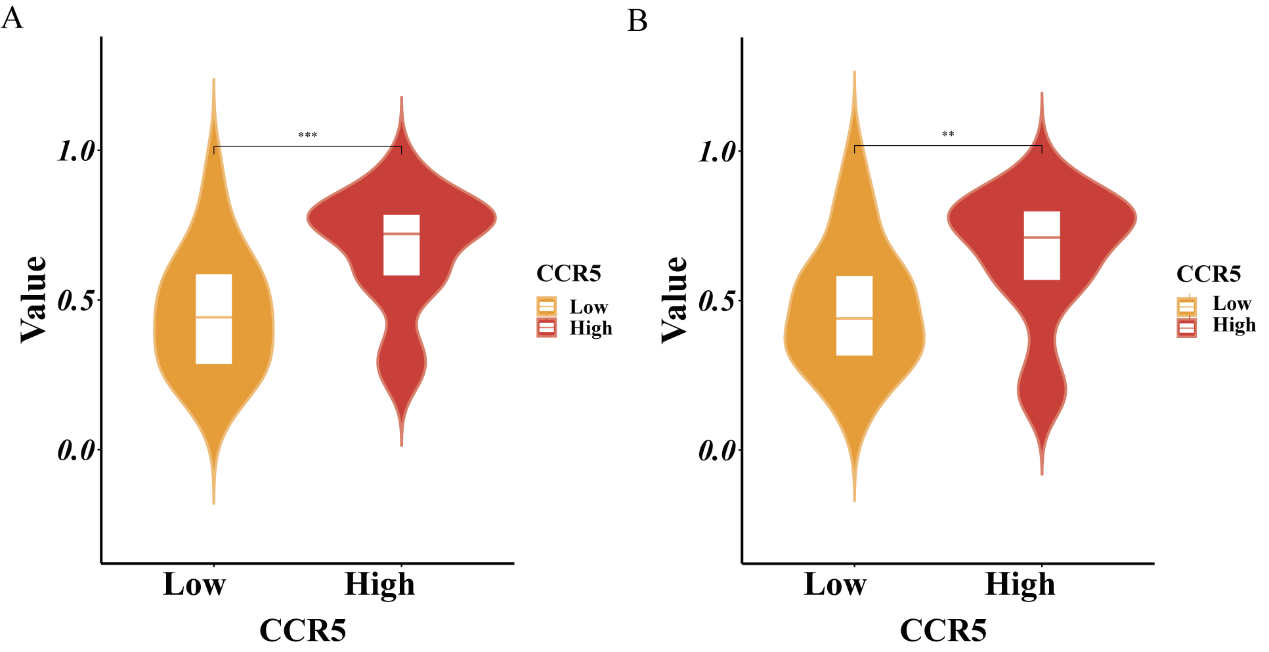


**Supplemental Figure 8: Principal Component Analysis (PCA) performed on the extracted features to plot data in a space of reduced dimensions.** Radiomics features of the two devices were similar.


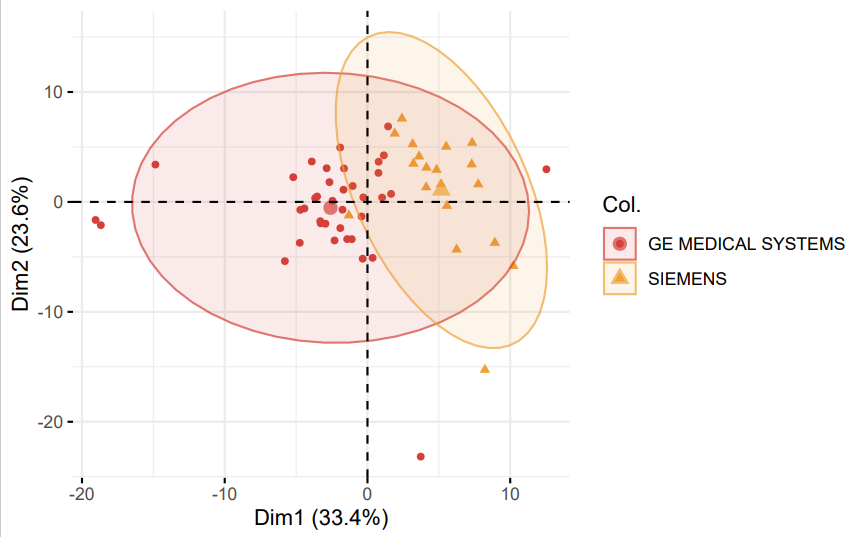

Supplement: Supplementary file 1 — Additional file 1: Supplemental Table 1. Inclusion/exclusion criteria applied to determine the study samples. Supplemental Table 2. Formula of the Radiomics model. Supplemental Table 3. AUC of the time-dependent ROC with different predictor. Supplemental Table 4. The image processing and feature extraction. Supplemental Figure 1. The image processing and feature extraction. Supplemental Figure 2. Relationship between CCR5 expression level and abundance of immune infiltrates in the study cohort. Supplemental Figure 3. GO and KEGG enrichment analysis of the CCR5. Supplemental Figure 4. Feature Selection of the radiomic model. Supplemental Figure 5. Comparison of the four optimal radiomic features between high-expression and low-expression CCR5 group. Supplemental Figure 6. Radiomic features' importance according to Logistic regression. Supplemental Figure 7. Difference between radiomics score when compared using Wilcoxon test in the training (A)and validation (B) set. Supplemental Figure 8. Principal Component Analysis (PCA) performed on the extracted features to plot data in a space of reduced dimensions. [file 13048_2022_1089_MOESM1_ESM.docx]
